# Transglutaminase 2 mediates UV-induced skin inflammation by enhancing inflammatory cytokine production

**DOI:** 10.1038/cddis.2017.550

**Published:** 2017-10-26

**Authors:** Seok-Jin Lee, Ki Baek Lee, Young Hoon Son, Jiwoong Shin, Jin-Haeng Lee, Hyo-Jun Kim, Ah-Young Hong, Hee Won Bae, Mee-ae Kwon, Won Jong Lee, Jin-Hee Kim, Dong Hun Lee, Eui Man Jeong, In-Gyu Kim

**Affiliations:** 1 Department of Biochemistry and Molecular Biology, Seoul National University College of Medicine, Seoul, Republic of Korea; 2 Department of Biomedical Laboratory Science, Cheongju University College of Health Science, Cheongju, Republic of Korea; 3 Department of Dermatology, Seoul National University College of Medicine, Seoul, Republic of Korea; 4 Institute of Human-Environment Interface Biology, Seoul National University College of Medicine, Seoul, Republic of Korea

## Abstract

UV irradiation elicits acute inflammation in the skin by increasing proinflammatory cytokine production in keratinocytes. However, the downstream protein target(s) that link UV radiation to the activation of signaling pathways responsible for cytokine expression have not been fully elucidated. In this study, we report a novel role of transglutaminase 2 (TG2), a member of the TG enzyme family whose activities are critical for cornified envelope formation, in mediating UV-induced inflammation. Our results showed that TG2-deficient mice exhibited reduced inflammatory responses to UV irradiation, including reduced erythema, edema, dilation of blood vessels, inflammatory cell infiltration, and levels of inflammatory cytokines. Using primary mouse keratinocytes and HaCaT cells, we found that UV irradiation-induced cytokine production by activating TG2, but not by upregulating TG2 expression, and that ER calcium release triggered by the UV-induced activation of phospholipase C was required for TG2 activation. Moreover, TG2 activity enhanced p65 phosphorylation, leading to an increase in NF-*κ*B transcriptional activity. These results indicate that TG2 is a critical mediator of cytokine expression in the UV-induced inflammatory response of keratinocytes, and suggest that TG2 inhibition might be useful for preventing UV-related skin disorders, such as photoaging and skin cancer caused by chronic UV exposure.

Transglutaminases (TGs) are a family of calcium-dependent enzymes that produce crosslinked, polyaminated, or deamidated proteins by catalyzing the acyl-transfer reaction between the glutamine and lysine residues of substrate proteins or polyamines.^1^ Several proteins are crosslinked by TGs, such as loricrin, involucrin, cystatin A, filaggrin, trichohyalin, small proline-rich proteins, and keratins, which are components of the cornified envelope (CE), a barrier structure found in terminally differentiated keratinocytes.^2^ In the skin, six isozymes among nine members of the TG family are expressed: TG1–TG5 and factor XIIIa.^3^ As TG isozymes share a common active-site structure and catalytic mechanism, isozyme-specific functions in the skin have been elucidated only by using isozyme-null mice and genetic analyses of congenital skin diseases.

TG1-null mice displayed defective CE formation in their stratum corneum and impaired skin barrier function, leading to death within 4–5 h after birth due to dehydration.^4^ Moreover, TG1 mutations are the most common cause of autosomal recessive congenital ichthyosis in humans,^5^ indicating that the crosslinking activity of TG1 plays a crucial role in epidermal barrier formation. Mice lacking TG3 showed thinner hair with markedly decreased crosslinking of hair proteins,^6^ and showed increased contact hypersensitivity elicited by FITC sensitization^7^ and increased apoptosis induced by UV radiation.^8^ Mutations in TG3 have been associated with uncombable hair syndrome,^9^ indicating that TG3 contributes to hair development as well as CE formation. TG5 is expressed in the upper layer of the human epidermis,^2, 10^ and TG5 mutations cause acral peeling skin syndrome in humans,^11^ indicating the role of TG5 in cell-to-cell adhesion in the uppermost layer of epidermis. TG4 is expressed in glandular epithelia but not in epidermal keratinocytes, and TG4-deficient mice showed no altered skin phenotypes.^12^ Factor XIIIa-deficient mice showed delayed and defective wound healing, indicating that the crosslinking activity of factor XIIIa plays a role in wound repair and remodeling.^13^ Unlike these TG isozymes, TG2-null mice showed no obvious altered skin phenotypes, despite their expression in epidermal keratinocytes.^14^ Moreover, there is no report of human skin disease associated with TG2. Thus, the role of TG2 in skin pathophysiology remains unknown.

UV irradiation causes acute inflammatory responses in the skin by inducing the synthesis and release of proinflammatory cytokines, such as tumor necrosis factor-*α* (TNF-*α*) and IL-6, from keratinocytes.^15^ Mechanistically, UV radiation generates reactive oxygen species (ROS) and DNA damage in keratinocytes, which activate the NF-*κ*B signaling pathway responsible for cytokine release.^16, 17^ However, the mechanism linking UV radiation with the activation of NF-*κ*B signaling remains unclear. Previously, we showed that intracellular TG2 is normally inactive, but activated under various stress conditions, including UV radiation, oxidative stress, hypoxia, and DNA damage, through the mobilization of calcium.^18, 19, 20, 21, 22^ In cultured cells, stress-induced TG2 activation results in the enhancement of cell survival by crosslinking I*κ*B and caspase-3.^19, 23^ In mouse models, the activation of TG2 is reportedly involved in the development of cataract formation, tissue injury-induced lung fibrosis, and resistance to cancer chemotherapy by aberrantly increasing crosslinked proteins.^1^ These findings indicate that TG2 functions as a stress responsive enzyme, modulating cellular functions through the modification of various substrate proteins.^1^

Herein, we determined whether TG2-mediated inflammatory responses in the UV-exposed skin, and showed that UV-induced TG2 activation is crucial for the NF-*κ*B-dependent expression of proinflammatory cytokines. In this process, PLC/IP_3_/IP_3_R-mediated ER calcium release acts as an upstream signal for the activation of TG2. These results indicate that TG2 is a critical regulator that mediates acute cutaneous inflammation in response to UV radiation.

## Results

### TG2-deficient mice show reduced UV-induced skin inflammation

**Figure 1:**
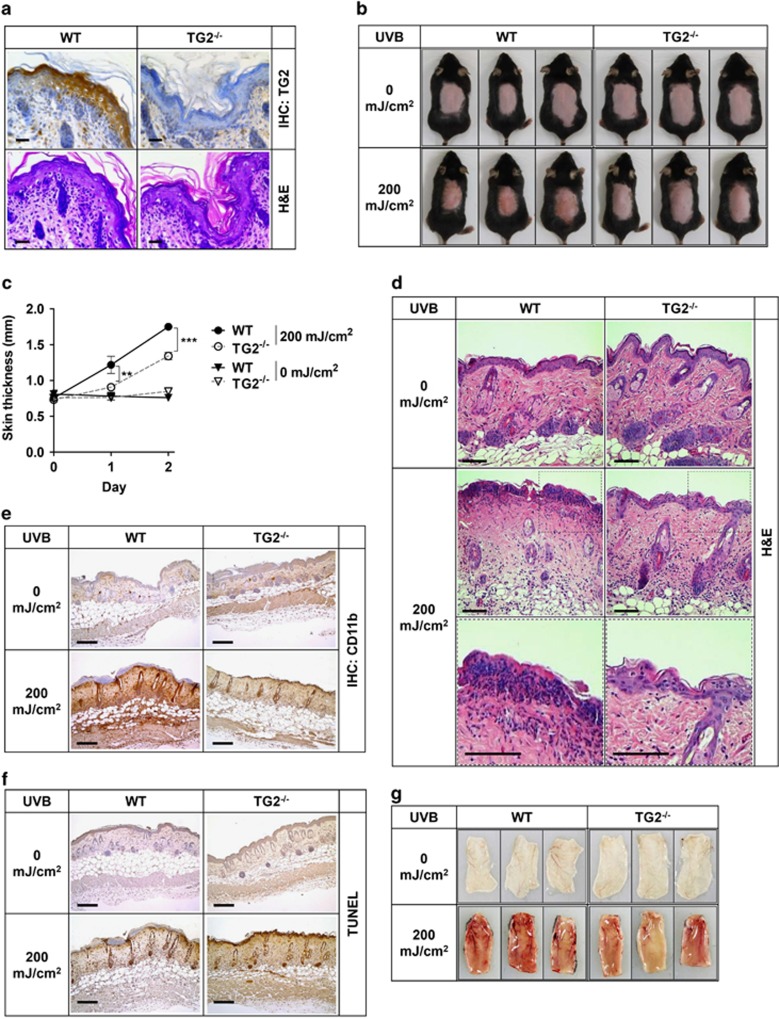
TG2^−/−^ mice exhibit reduced skin inflammation in response to UV irradiation. (**a**) Histology of the skin from WT and TG2^−/−^ littermates (8-week old, female). Skin sections were stained with anti-TG2 antibody and hematoxylin & eosin (H&E). (**b**) Representative photographs of mice skin after 48 h of UV exposure (200 mJ/cm^2^ UVB). (**c**) Skin thickness measured by caliper at indicated times. Data are shown as mean±SEM. (**d**–**f**) Histology and immunohistochemistry of the skin from WT and TG2^−/−^ littermates after 48 h of UV irradiation (200 mJ/cm^2^ UVB). Skin sections were stained with H&E (**d**), CD11b (**e**), and TUNEL (**f**). (**g**) Vasculature of the UV-irradiated skin after 96 h of UV exposure. Scale bars, 100 *μ*m. *P<0.05; **P<0.01; ***P<0.001

The crosslinking activity of TG1 and TG3 is crucial for CE formation during the terminal differentiation of epidermal keratinocytes.^2^ To test whether TG2 is also involved in this process, we examined the skin barrier function of TG2^−/−^ mice. In wild-type (WT) mice, TG2 is expressed in all epidermal layers (Figure 1a). The histochemical staining of skin sections showed no differences between WT and TG2^−/−^ mice in gross structure of the skin and the expression of differentiation markers, such as loricrin, involucrin, and filaggrin in epidermal keratinocytes (Figure 1a and Supplementary Figure 1A). The functional evaluation of skin permeability with toluidine blue staining revealed no difference between WT and TG2^−/−^ mice (Supplementary Figure 1B). These results indicate that TG2 activity is not required for the differentiation or CE formation of epidermal keratinocytes.

TG2 is activated under various ROS-producing conditions, including UV irradiation.^21^ To determine whether TG2 is involved in UV-induced skin inflammation, we compared the effects of UV irradiation on the skin of WT and TG2^−/−^ mice. After 48 h of UVB irradiation, TG2^−/−^ mice showed reduced skin redness (Figure 1b), significantly decreased swelling of the skin (Figure 1c), reduced infiltration of immune cells to the UV-irradiated skin (Figures 1d and e), and reduced vasodilation in UV-irradiated lesions (Figure 1g) compared with those of WT littermates. These skin phenotypes of TG2^−/−^ mice suggest a causal role of TG2 in UV-induced acute inflammation. On the other hand, UV-irradiated WT and TG2^−/−^ mice exhibited no significant difference in the number of TUNEL-positive skin cells (Figure 1f), indicating that TG2 may not be involved in UV-induced skin cell apoptosis.

### TG2 mediates the UV-induced production of inflammatory cytokines in keratinocytes

**Figure 2:**
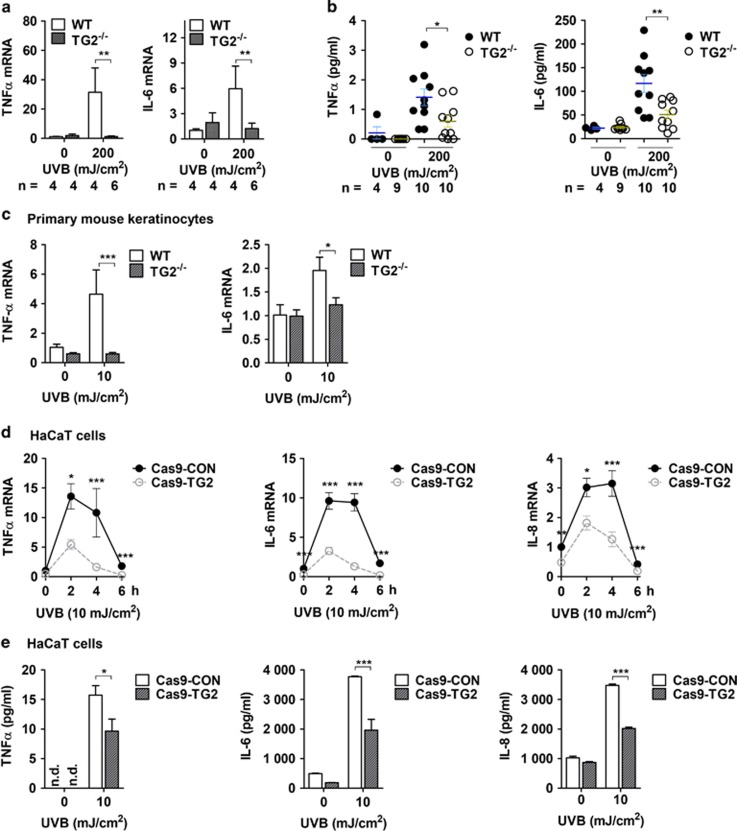
TG2 is required for the expression of inflammatory cytokines in UV-irradiated keratinocytes. (**a** and **b**) Skin tissues were obtained from WT and TG2^−/−^ littermates after 48 h of UV irradiation (200 mJ/cm^2^). Levels of mRNA (**a**) and protein (**b**) for mouse IL-6 and TNF-*α* were determined by QRT-PCR and MAGPIX Multiplexing assay, respectively. (**c**) Primary mouse keratinocytes from WT and TG2^−/−^ littermates were cultured and exposed to UV irradiation (10 mJ/cm^2^). After 6 h, mRNA levels of mIL-6 and mTNF-*α* were determined by QRT-PCR (*n*=3). (**d** and **e**) TG2-deficient HaCaT cells were generated by using the CRISPR–Cas9 system (Cas9-TG2) and exposed to UV irradiation (10 mJ/cm^2^). After 6 h, levels of mRNA (**d**, *n*=3) and secreted protein (**e**, *n*=3) for TNF-*α*, IL-6, and IL-8 were measured by QRT-PCR and by the CBA method, respectively. n.d., not determined. All data are represented as mean±SEM. *P<0.05; **P<0.01; ***P<0.001

UV irradiation stimulates the production and release of inflammatory cytokines in epidermal keratinocytes, which elicits skin inflammation. To test whether TG2 mediates UV-induced cytokine production, we compared the ability of WT and TG2^−/−^ mice to produce inflammatory cytokines in response to UV irradiation. In the UV-exposed skin from WT mice, there was a 30- and 6-fold increase in the mRNA levels of TNF-*α* and IL-6, respectively, whereas there was no change in the expression levels of both cytokines in the skin of TG2^−/−^ mice (Figure 2a). MAGPIX multiplexing revealed that the protein levels of both TNF-*α* and IL-6 were significantly reduced in TG2^−/−^ mice compared with those in the skin of WT mice in response to UV irradiation (Figure 2b). To confirm the role of TG2 in keratinocytes, we prepared mouse neonatal epidermal keratinocytes (MNEKs) and examined the TNF-*α* and IL-6 expression levels. MNEKs from TG2^−/−^ mice showed significantly reduced levels of TNF-*α* and IL-6 mRNA after UVB exposure compared with those from WT mice (Figure 2c).

To test the role of TG2 in human keratinocytes, we generated TG2^−/−^ and knockdown HaCaT cell lines by using the CRISPR–Cas9 system and small hairpin RNA transfection, respectively, and compared the levels of cytokine expression. In control HaCaT cells, the mRNA levels of TNF-*α*, IL-6, and IL-8 rapidly increased after UV irradiation, remained constant for 2–4 h, and decreased thereafter to basal levels. In contrast, in TG2^−/−^ and knockdown HaCaT cells, the mRNA levels were significantly suppressed (Figure 2d and Supplementary Figure 2A). The protein levels of TNF-*α*, IL-6, and IL-8 secreted from UV-irradiated HaCaT cells were also significantly reduced in TG2^−/−^ and knockdown cells (Figure 2e and Supplementary Figure 2B online). These results indicate that UV irradiation induces the production of inflammatory cytokines through the regulation of TG2 in epidermal keratinocytes.

### UV irradiation increases TG2 activity but not its protein level

**Figure 3:**
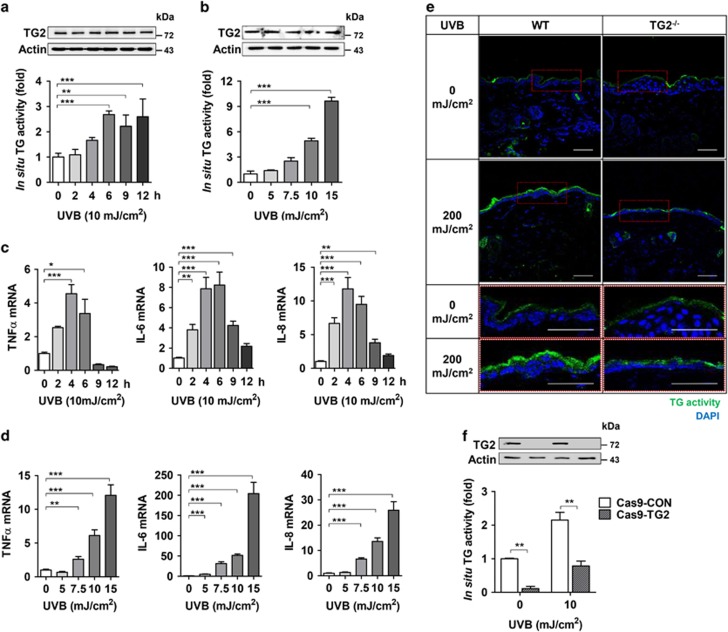
UV irradiation activates keratinocyte TG2. (**a** and **b**) Levels of TG2 protein and *in situ* TG activity in HaCaT cells were determined by western blot analysis and biotinylated pentylamine (BP) incorporation assay, respectively. HaCaT cells were exposed to UV irradiation (**a**, 10 mJ/cm^2^, *n*=4; **b**, doses indicated, *n*=4) and collected at the time indicated (**a**) or after 6 h of UV irradiation (**b**). (**c** and **d**) mRNA levels of TNF-*α*, IL-6, and IL-8 were determined by QRT-PCR in HaCaT cells exposed to UV irradiation as (**a**) and (**b**) (*n*=3). (**e**) *In situ* TG activity in the skin from UV-irradiated WT and TG2^−/−^ littermates (*n*=3). (**f**) *In situ* TG activity in TG2-deficient HaCaT cells (Cas9–TG2) after 6 h of UV irradiation (10 mJ/cm^2^). All data are represented as mean±SEM. *P<0.05; **P<0.01; ***P<0.001

To gain evidence that TG2 is regulated by UV irradiation, we monitored the TG2 protein level and intracellular transamidation activity in UV-exposed HaCaT cells. Western blot analysis showed that there were no changes in the protein level of TG2 for 12 h after UV irradiation (Figure 3a), even with increased dosage (Figure 3b). Moreover, the mRNA levels of TG2 were not affected by UV irradiation (Supplementary Figure 3). By contrast, UV irradiation induced a time- (Figure 3a) and dose-dependent (Figure 3b) increase of *in situ* TG activity, which paralleled the mRNA levels of TNF-*α*, IL-6, and IL-8 in a time-dependent (Figure 3c) and dose-dependent manner (Figure 3d), indicating that UV-induced cytokine production is correlated with *in situ* TG activity but not with the TG2 protein level.

In addition to TG2, TG1, 3, and 5 are also expressed in epidermal keratinocytes,^2^ and the current TG assay method is not able to discriminate TG isoenzymes. To test whether UV irradiation activates TG2 only, we compared *in situ* TG activity in the skin of TG2^−/−^ mice with that of WT mice. TG2^−/−^ mice showed no increase in TG activity in the epidermis in response to UV irradiation, whereas WT mice exhibited substantially increased TG activity (Figure 3e). Similar results were observed in the TG2-deficient HaCaT cell line (Figure 3f), indicating that an increase in *in situ* TG activity can be attributed to UV-induced TG2 activation.

**Figure 4:**
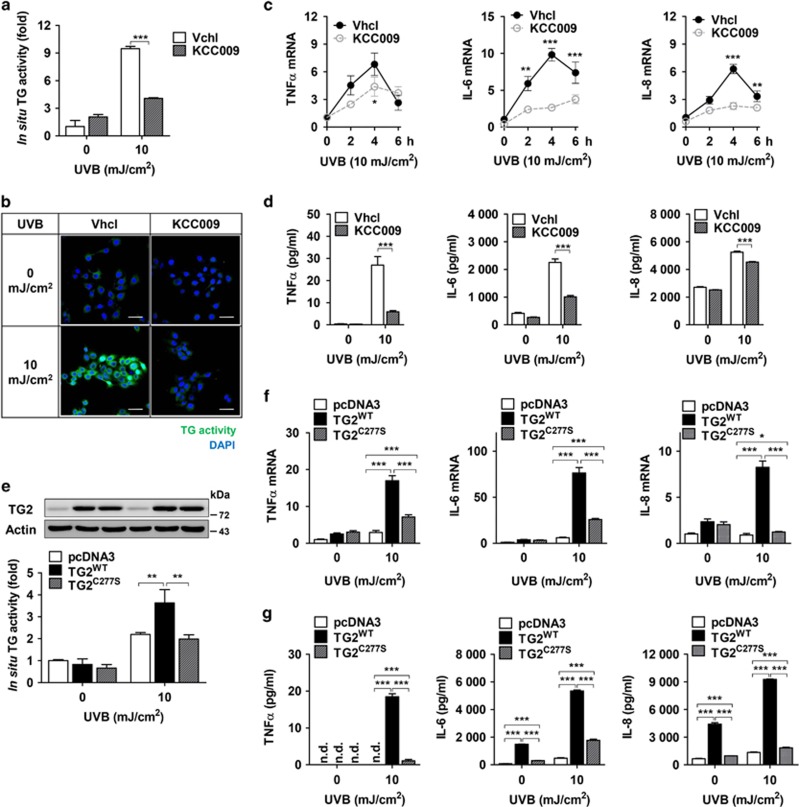
TG inhibition suppresses the production of cytokines in UV-irradiated keratinocytes. (**a** and **b**) HaCaT cells were pretreated with KCC009 (250 *μ*M) or vehicle (Vhcl) 1 h before UV irradiation (10 mJ/cm^2^). The cells were incubated for 6 h after UV irradiation in the same media. *In situ* TG activity was measured by the BP incorporation assay (**a**, *n*=3) and visualized by confocal microscopy (**b**). Scale bars, 50 *μ*m. (**c** and **d**) Levels of mRNA (**c**, *n*=3) and proteins (**d**, *n*=3) for TNF-*α*, IL-6, and IL-8 were measured in KCC009-pretreated and UV-irradiated HaCaT cells at the times indicated (**c**) or 6 h after UV irradiation (**d**). (**e**) Levels of TG2 protein and *in situ* TG activity were measured in HaCaT cell lines expressing pcDNA3, WT (TG2^WT^), or active-site mutant TG2 (TG2^C277S^) established by transfection and selection (*n*=4). (**f** and **g**) Levels of mRNA (**f**, *n*=4) and proteins (**g**, *n*=4) for TNF-*α*, IL-6, and IL-8 were measured in HaCaT cell lines expressing pcDNA3, WT (TG2^WT^) or active-site mutant TG2 (TG2^C277S^) at 6 h after UV irradiation. n.d., not determined. All data are represented as mean±SEM. *P<0.05; **P<0.01; ***P<0.001

To confirm these results, we examined the effect of KCC009, an inhibitor of TG,^24^ on UV-induced cytokine expression. In HaCaT cells exposed to UV irradiation, KCC009 treatment significantly suppressed *in situ* TG activity (Figures 4a and b), and concomitantly reduced the mRNA (Figure 4c) and protein levels of TNF-*α*, IL-6, and IL-8 compared with control cells (Figure 4d). To further confirm TG2-specific activation by UV irradiation, we established HaCaT cell lines overexpressing WT TG2 (TG2^WT^) or active-site mutant of TG2 (TG2^C277S^). When these cells were exposed to UV irradiation, *in situ* TG activity was increased in proportion to TG2 protein levels. By contrast, HaCaT cells expressing TG2^C277S^ failed to increase *in situ* TG activity (Figure 4e), mRNA levels (Figure 4f), and protein levels of proinflammatory cytokines compared with cells overexpressing TG2^WT^ (Figure 4g). These results indicate that UV irradiation promotes cytokine production through TG2 activation, but not through the upregulation of TG2 expression.

### UV irradiation activates TG2 through endoplasmic reticulum (ER) calcium release

**Figure 5:**
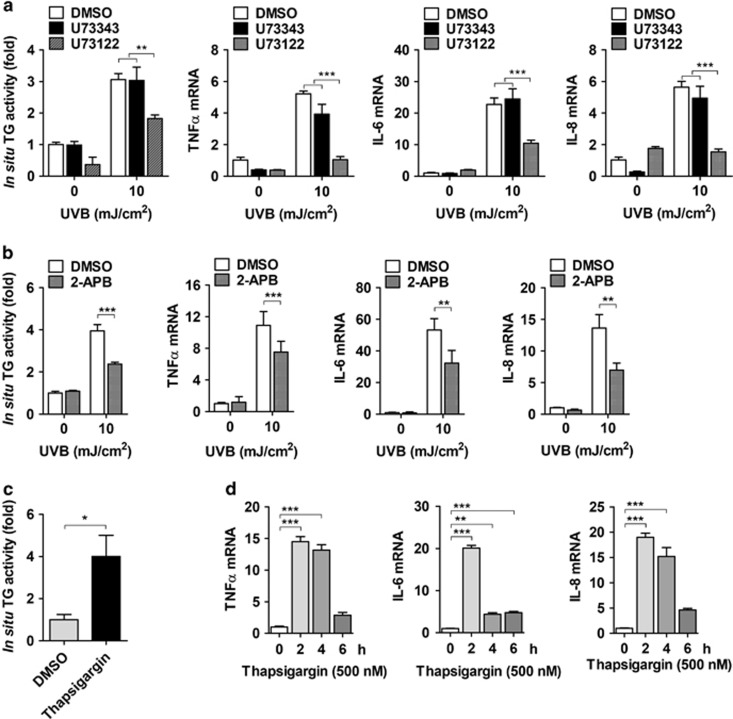
UV-induced release of ER calcium is responsible for TG2 activation. (**a** and **b**) HaCaT cells were pretreated with 20 *μ*M U73122 or U73343 (**a**, *n*=3) and with 100 *μ*M 2-aminoethyl diphenylborinate (2-APB) (**b**, *n*=3), and then exposed to UV irradiation (10 mJ/cm^2^). After 6 h, levels of *in situ* TG activity and mRNA for TNF-*α*, IL-6, and IL-8 were measured by the BP incorporation assay and QRT-PCR, respectively. (**c** and **d**) Effect of thapsigargin (500 nM) on *in situ* TG activity in HaCaT cells (**c**, *n*=3) and the expression of cytokines (**d**, *n*=3) or in primary epidermal keratinocytes prepared from WT and TG2^−/−^ littermates. All data are represented as mean±SEM. *P<0.05; **P<0.01; ***P<0.001

We next investigated the mechanism by which UV irradiation activates TG2 in epidermal keratinocytes. As TG2 is a calcium-dependent enzyme, we considered the role of calcium in activating TG2 in UV-irradiated keratinocytes. UV irradiation increases cytosolic Ca^2+^, which is released from the ER through the activation of the PLC-IP_3_ signaling pathway.^25^ To determine whether PLC activation is required for the UV-induced increase of TG2 activity, we examined the effect of U73122, a pan-PLC inhibitor, on *in situ* TG activity. In HaCaT cells exposed to UV irradiation, pretreatment with U73122 suppressed *in situ* TG activity and the mRNA expression of TNF-*α*, IL-6, and IL-8, whereas pretreatment with its inactive analogue U73343 showed no effect (Figure 5a). Treatment with 2-aminoethyl diphenylborinate, an IP_3_R inhibitor, also exhibited suppressed *in situ* TG activity and cytokine mRNA expression (Figure 5b). To confirm the role of ER calcium in activating TG2, we evaluated the effect of thapsigargin treatment in HaCaT cells, which increased *in situ* TG activity and the mRNA levels of inflammatory cytokines (Figures 5c and d). These results indicate that TG2 is activated by the UV-induced release of ER calcium through the PLC-IP_3_ signaling pathway.

### Activation of TG2 enhances NF-*κ*B transcriptional activity

**Figure 6:**
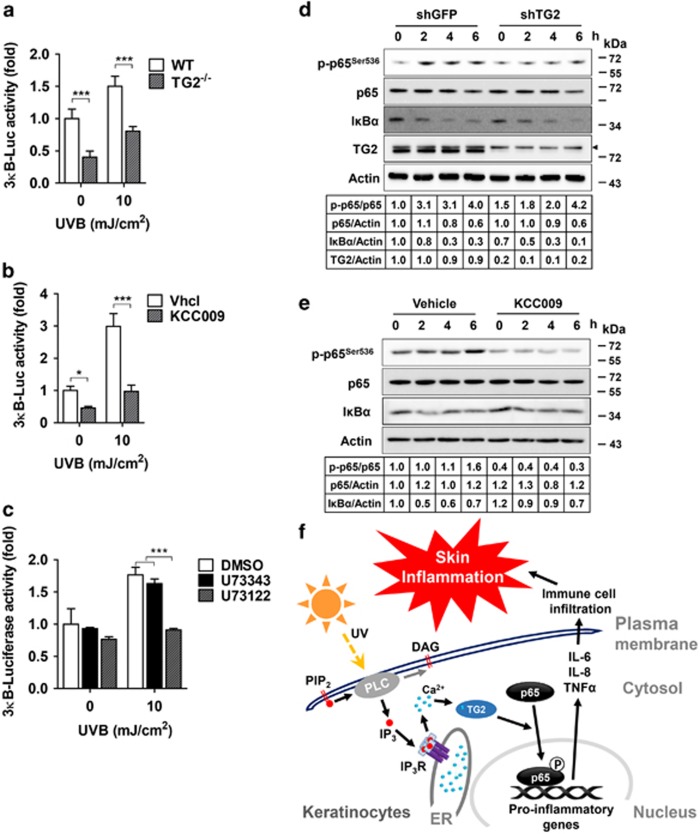
TG2 enhances the transcriptional activity of NF-*κ*B. (**a**–**c**) 3*κ*B-luciferase reporter activity was assessed in primary epidermal keratinocytes prepared from WT and TG2^−/−^ littermates (**a**, *n*=3) or HaCaT cells (**b** and **c**; *n*=3) 9 h after UV irradiation (10 mJ/cm^2^). HaCaT cells were pretreated with 250 *μ*M KCC009 (**b**), or with 20 *μ*M U73122 or U73343 (**c**) 1 h before UV irradiation. Luciferase activity was normalized with co-transfected *Renilla* activity. Data are represented as mean±SEM. *P<0.05; **P<0.01; ***P<0.001. (**d** and **e**) Lysates prepared from TG2 knockdown HaCaT cells (**d**) or HaCaT cells treated with 250 *μ*M KCC009 (**e**) were immunoblotted with antibodies specific for I*κ*B*α*, phosphorylated p65 at Ser536, p65, and TG2. Actin was used as a loading control. An arrowhead in the blot indicates a nonspecific band. (**f**) Schematic representation of TG2-dependent UV-induced inflammation

The NF-*κ*B signaling pathway plays a major role in the production of proinflammatory cytokines in response to UV irradiation,^26^ and TG2 is known to activate NF-*κ*B signaling under oxidative stress; however, its molecular mechanism remains unclear.^23, 27^ To determine whether TG2 is involved in NF-*κ*B activation under UV-irradiated conditions, we compared the ability to promote the transcriptional activity of NF-*κ*B in WT and TG2^−/−^ MNEKs transiently transfected with the 3*κ*B-luciferase gene. TG2^−/−^ MNEKs showed significantly reduced NF-*κ*B reporter activity compared with WT MNEKs after UV irradiation (Figure 6a). In HaCaT cells stably expressing 3*κ*B-luciferase, treatment with KCC009 or U73122 significantly inhibited the UV-induced reporter activity of NF-*κ*B (Figures 6b and c), indicating that TG2 activity is required for UV-induced NF-*κ*B activation.

To further analyze TG2-mediated NF-*κ*B activation, we monitored the protein levels of I*κ*B*α* and phosphorylated p65. Western blot analysis showed that I*κ*B*α* levels were decreased after UV irradiation in a time-dependent manner in TG2 knockdown, KCC009-treated, and control HaCaT cells. By contrast, the levels of phosphorylated p65 (p-p65^Ser536^) were increased in control HaCaT cells, but decreased in TG2 knockdown and KCC009-treated cells (Figures 6d and e), indicating that TG2 mediates NF-*κ*B activation not by inducing I*κ*B*α* degradation, but by promoting the phosphorylation of p65 at Ser^536^. These results indicate that UV irradiation activates TG2 through the PLC-IP_3_ pathway, which activates NF-*κ*B signaling by promoting p65 phosphorylation, leading to an increase in proinflammatory cytokine production (Figure 6f).

## Discussion

UV irradiation elicits a range of responses in the skin, including inflammatory and DNA damaging responses, which lead to the development of photoaging and skin cancer in chronically exposed skin.^16^ Of these, acute inflammatory reaction is mediated by the UV-induced release of proinflammatory cytokines, such as TNF-*α*, IL-6, and IL-8. However, the signaling pathways responsible for cytokine production in UV-exposed keratinocytes have not been fully characterized. Herein, we show that keratinocyte TG2 is a critical mediator in UV-induced acute skin inflammation, based on a phenotype analysis of TG2-deficient mice. Moreover, our results identify the PLC-IP_3_ pathway as an upstream pathway for UV-induced TG2 activation and the NF-*κ*B pathway as a downstream pathway for the TG2-induced release of proinflammatory cytokines. Collectively, these results indicate that TG2 is an effector enzyme that regulates NF-*κ*B activity following UV irradiation in epidermal keratinocytes.

Skin barrier function and keratinization were impaired in autosomal recessive congenital ichthyosis 1 caused by TG1 mutation,^4, 5^ and skin barrier defects observed in TG3^−/−^ mice caused the reduction of inflammatory threshold of the skin exposed to UV irradiation.^7^ These observations indicate that the crosslinking activity of TG1 and TG3 in epidermal keratinocytes plays a critical role in the formation of the skin barrier, which in turn affects the inflammatory response to UV irradiation. In contrast, our data show that keratinization and skin barrier function were normal in TG2^−/−^ mice. Consistent with these results, recent reports have shown no difference between TG1^−/−^ and TG1/TG2 double knockout mice in the structure of the epidermal CE.^28^ Thus, our results indicate that the reduced inflammatory response of TG2^−/−^ mice to UV irradiation is not attributable to the change in barrier function.

Our results provide insight into the role of PLC signaling in skin inflammation. PLC regulates cellular functions through the hydrolysis of PIP_2_ to DAG and IP_3_, which binds with IP_3_R on the ER membrane, leading to the release of ER calcium into the cytosol.^29^ In keratinocytes, PLC is a known regulator of inflammation. PLC*ε*-knockout mice exhibited reduced UV-induced skin inflammation,^30, 31^ and reduced allergic contact hypersensitivity due to the hampered expression of proinflammatory cytokines.^32^ Moreover, transgenic mice overexpressing PLC*ε* in epidermal keratinocytes showed the development of spontaneous skin inflammation.^33^ However, the pathway that mediates PLC-induced inflammation remains unknown. Our results demonstrate that TG2 is activated by an increase in cytosolic calcium and it modulates NF-*κ*B activity, thus indicating a pathway linking PLC signaling with skin inflammation. UV irradiation is also known to induce ER stress, triggering unfolded protein response by the release of ER calcium.^25, 34^ Consistent with these findings, we showed that TG2 is activated by ER stress via oxidative stress-induced ER calcium release.^20^ Moreover, a recent report showed that TG2 crosslinks IP_3_R-1, thereby inhibiting ER calcium release.^35^ This reaction might be a negative feedback mechanism to maintain intracellular calcium homeostasis and TG2 activity. These results imply that the regulation of TG2 activity by ER calcium is a critical point of control in the inflammatory response to UV irradiation.

TG2 is a calcium-dependent enzyme, and it is known that the EC_50_ of [Ca^2+^]_i_ required for the activation of TG2 is ~100–500 *μ*M.^36, 37, 38, 39, 40^ At these calcium levels, TG isoenzymes expressed in epidermal keratinocytes, such as TG1 and TG3, can also be activated. As it is not possible to evaluate TG isoenzyme-specific activity due to the lack of an effective assay method and an isoenzyme-specific inhibitor, we used a genetic approach to assess UV-induced TG activation. Our results show that TG2 contributes >90% of basal and 60% of UV-induced TG activity by comparison of *in situ* TG activity between WT and TG2-deficient HaCaT cells (Figure 3f). Moreover, our data demonstrate that a majorly activated TG isotype in the UV-irradiated skin is TG2 in the upper layers of epidermis, where TG1 and TG3 are expressed, as well as in the lower layers by comparison of *in situ* TG activity between WT and TG2^−/−^ mice (Figure 3e). These results indicate that although TG1 and TG3 are also activated by UV irradiation, TG2 activation may account for most of the UV-induced increase of epidermal TG activity. In support of these data, proteolytic cleavage is required for TG1 and TG3 activation, in addition to an increase in [Ca^2+^]_i_ during the terminal differentiation of keratinocytes.^41, 42^ Nevertheless, UV irradiation upregulates TG1 expression through the NF-*κ*B pathway,^43^ leading to the restoration of damaged barrier function.^44^ TG3 has a protective role against photodamage, owing to the requirement of its activity in CE assembly.^8^ Thus, TG1 or TG3 inactivation might exacerbate UV-induced skin inflammation.

TG2 positively regulates NF-*κ*B signaling in various cancer cell lines. However, the substrate protein(s), whose function is altered by TG2-mediated modification, remains unidentified. Here, we tested two proposed mechanisms for the activation of NF-*κ*B signaling. First, TG2 is known to activate NF-*κ*B through the crosslinking of I*κ*B*α*, which induces the release and subsequent nuclear translocation of NF-*κ*B.^23^ However, our results show that the protein level of monomeric I*κ*B*α* was decreased by UV irradiation, even in TG2 knockdown, inhibitor-treated, and control HaCaT cells. Moreover, dimeric or multimeric forms of crosslinked I*κ*B*α* were not observed in the same experimental conditions, indicating that I*κ*B*α* crosslinking did not occur in TG2-mediated NF-*κ*B activation. Second, TG2 reportedly facilitates the nuclear translocation of NF-*κ*B, regardless of its enzymatic activity in breast cancer cells.^27^ In contrast to this report, our data show that the expression of enzymatically inactive TG2 failed to increase the expression of proinflammatory cytokines after UV irradiation, demonstrating that the transamidation activity of TG2 is required for UV-induced NF-*κ*B activation. Instead, we show that p65^Ser536^ phosphorylation was increased, but decreased in TG2 knockdown or inhibitor-treated cells after UV irradiation (Figures 6d and e). As Ser536 is phosphorylated by IKK^45^ and is a functionally important residue in regulating the transcriptional activity of p65,^46^ our results imply that TG2 activates NF-*κ*B signaling by increasing p65^Ser536^ phosphorylation in UV-irradiated keratinocytes. However, there is no evidence that TG2 regulates the kinase activity of IKKs. Moreover, it was reported that TG2 activates NF-*κ*B signaling in an IKK-independent manner.^47^ Thus, further investigation of the molecular mechanism by which TG2 regulates the phosphorylation of p65^Ser536^ is needed.

In summary, we have shown that keratinocyte TG2 plays a pivotal role in mediating UV-induced skin inflammation. TG2 is activated by UV-induced ER calcium release through the PLC-IP_3_ signaling pathway, which in turn activates NF-*κ*B signaling, leading to an increase in proinflammatory cytokine production (Figure 6f). These findings suggest that the inhibition of keratinocyte TG2 might be a useful strategy for the prevention of UV radiation-related skin disorders, such as photoaging and skin cancer, which occur with chronic exposure to UV irradiation.

## Materials and methods

### Mice

TG2^−/−^ mice^14^ were backcrossed 12 times on a C57BL/6J background. Female WT and TG2^−/−^ littermates (8-week old) were used for all experiments. All mice were bled and kept in our animal facility at the Seoul National University College of Medicine, Seoul, South Korea, under standard conditions (22±1 °C, 55±5% humidity, 12-h light and 12-h dark cycle). Animal experiments were approved by the Seoul National University Institutional Animal Care and Use Committee (IACUC No. SNU-130103-1-5). The dorsal skin was shaved and chemically depilated by Veet cream (Oxy-Reckitt Benckiser, Slough, UK) 48 h before UV irradiation.

### Immunohistochemical analysis

Mouse skin tissues were fixed in 4% paraformaldehyde for 24 h, embedded in paraffin, and processed to get 4 *μ*m thick sections for hematoxylin and eosin staining or immunohistochemical analysis. The sections were dewaxed in xylene (Junsei Chemical Co., Ltd, Tokyo, Japan), unmasked by heat-mediated antigen retrieval, and incubated in 0.3% H_2_O_2_ in methanol for 30 min at room temperature to eliminate endogenous peroxidase activity. The sections were then blocked for 60 min at room temperature in blocking serum in phosphate-buffered saline (PBS) and incubated with anti-TG2 antibody (Thermo Fisher Scientific Inc., Rockford, IL, USA, RB-060-P) or anti-CD11b antibody (Abcam, Cambridge, MA, USA, ab133357), followed by equilibration at 4 °C overnight. The next day, sections were incubated in each biotinylated secondary antibody for 30 min at room temperature. The secondary antibody was visualized using a Vectastain ABC Elite Reagent (Vector Laboratories Inc., Burlingame, CA, USA) and 3,3′-diaminobenzidine (Dako, Glostrup, Denmark) according to the manufacturer’s instructions. TUNEL staining was carried out according to the manufacturer’s protocol in the ‘ApopTag Peroxidase *In Situ* Apoptosis Detection Kit’ manual (Chemicon International, Inc., MA, USA). Mayer’s hematoxylin was used as a counterstain. Coverslips were mounted and sealed with Tissue Tek Glas Mounting Media (Sakura, Torrance, CA, USA). Bright-field imaging was performed using a Leica-microscope (Leica Microsystems, Bensheim, Germany), and the images were captured using Microscope Imaging Software (Leica Microsystems). For the *in situ* TG2 activity assay in mice exposed to UV irradiation (200 mJ/cm^2^), biotinylated pentylamine (BP) (0.1 mg/g) was administered 5 h after irradiation by intraperitoneal injection. Mice were killed 3 h later, and skin tissues were embedded in Tissue-TEK OCT compounds (Sakura), reacted with streptavidin-488 (Thermo Fisher Scientific Inc.) and visualized using a FluoView 1000 confocal microscope (Olympus, Tokyo, Japan).

### Cell culture

HaCaT cells were cultured in Dulbecco’s modified Eagle’s medium (DMEM, Welgene, Gyeongsan, South Korea) containing 10% heat-inactivated fetal bovine serum (Hyclone, Logan, UT, UK), 100 U/ml of penicillin, and 100 *μ*g/ml of streptomycin sulfate (Gibco, Carlsbad, CA, USA) under 5% CO_2_ at 37 °C. The TG2-deficient HaCaT cell line was generated by transfection with the TG2 CRISPR/Cas9 vector (Santa Cruz Biotechnologies, Santa Cruz, CA, USA) and sorted by FACS. HaCaT cells over-expressing TG2^WT^ or TG2^C277S^ were generated by transfection with WT or C277S mutant cDNA in the pcDNA vector, respectively. All cells were selected with G418 (1 mg/ml, Sigma-Aldrich, St Louis, MO, USA) for 1 week. All cell lines were regularly tested to exclude mycoplasma contamination.

MNEKs were prepared from neonatal skin as previously described with minor modifications.^48^ In brief, mice were killed with CO_2_ 2 days after birth. Mice were washed twice with Betadine and then rinsed with PBS. The skin of neonates was removed and soaked in a PluriSTEM Dispase-II solution (Merck Millipore, Darmstadt, Germany) for 12 h at 4 °C. The skin was then separated to epidermis and dermis. The epidermal layer was incubated in 1 ml of TrypLE (Gibco, Grand Island, NY, USA) for 10 min at 37 °C, and then 1 ml of fetal bovine serum was added to inactivate TrypLE. The epidermal tissues were vortexed for 10 min followed by centrifugation for 3 min at 1000 × *g* at room temperature. The cell pellet was resuspended in keratinocyte proliferation media (1 : 1 mixture of KGM-Gold and calcium free KGM-Gold kit media, Lonza, Lyon, France). MNEKs were used at passage 1.

### UV irradiation

A G20T10E UVB lamp (Sankyo, Denki, Japan) was used for UV irradiation. UVB was measured using a UV light meter, UV-340A (Lutron Electronic Enterprise Co. LTD, Taipei, Taiwan). Cells and mice were exposed to a single dose of UVB radiation (10 mJ/cm^2^ for cells and 200 mJ/cm^2^ for mice). The cells in a six-well plate were washed twice with PBS after the removal of culture media, 500 *μ*L of PBS was added, and they were exposed to UVB radiation.

### Western blot analysis

Sample preparation and SDS-PAGE were performed as previously described.^49^ The following primary antibodies were used: anti-*β*-actin (Sigma-Aldrich, A5441), TG2,^50^ p65 (Santa Cruz Biotechnology, Santa Cruz, CA, USA, sc-8008), p-p65^Ser536^ (Cell Signaling Technology, Beverly, MA, USA, #3031) and I*κ*B*α* (Cell Signaling Technology, sc-847). After the reaction with horseradish peroxidase-conjugated secondary antibody (Santa Cruz Biotechnology, sc-2004 or sc-2005), immunoreactive proteins were visualized by using a SuperSignal West Pico Chemiluminescent Substrate (Thermo Fisher Scientific Inc.). The bands were quantified using ImageJ (http://rsb.info.nih.gov/ij/).

### QRT-PCR

Total RNA extraction and QRT-PCR were carried out using a CFX96 Real-Time system (Bio-Rad, Hercules, CA, USA) as previously described.^49^ The following specific primers were used.


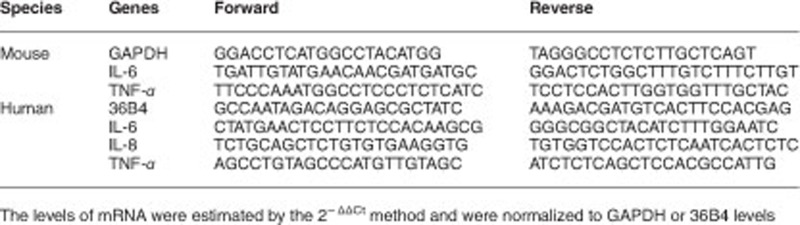


### Cytometric bead array

Secreted human TNF-*α*, IL-6, and -8 protein levels were measured by using a BD cytometric bead array (CBA) (Thermo Fisher Scientific Inc.). In brief, 4.5 × 10^5^ HaCaT cells were seeded on six-well plates and incubated for 24 h. Then, the culture medium was collected 6 h after UVB irradiation and analyzed according to the manufacturer’s instructions. The MAGPIX Multiplexing System (Komabiotech, Seoul, Korea) was used to measure the protein levels of mouse IL-6 and TNF-*α*.

### Luciferase reporter assay

HaCaT cells and MNEKs were co-transfected with the 3*κ*B-luciferase construct and pRL-TK vector as an internal control. Luciferase reporter activity was measured by using a luciferase assay kit (Promega, Madison, WI, USA) 9 h after UVB irradiation.

### *In situ* TG activity assay

Six hours after UVB irradiation, HaCaT cells were incubated with 1 mM EZ-link Pentylamine-Biotin (BP) (Thermo Fisher Scientific Inc.) as a TG2 substrate in serum-free DMEM for 1 h. These cells were subjected to the *in situ* TG activity assay as previously described.^19^

### Statistical analysis

Statistical evaluations were performed with GraphPad Prism 5.0 statistical software (GraphPad Software, La Jolla, CA, USA) using Student’s *t*-test, or one-way or two-way ANOVA. *P*<0.05 was considered statistically significant. All error bars represent mean±SEM. The data are representative of at least three independent experiments.

## Publisher’s Note

Springer Nature remains neutral with regard to jurisdictional claims in published maps and institutional affiliations.
