# Supplementary material for: Transglutaminase 2 mediates UV-induced skin inflammation by enhancing inflammatory cytokine production
Source: Cell Death Dis. 2017 Oct 26;8(10):e3148–. doi: 10.1038/cddis.2017.550 (PMC5680918; doi:10.1038/cddis.2017.550)
Supplement: Supplementary Information [file cddis2017550x1.pdf]

Supplementary Information

Supplementary Materials and Methods

shRNA expressing HaCaT cell lines

For generating short hairpin RNA (shRNA) expressing vectors, the following forward and reverse oligonucleotides were annealed in annealing buffer (100 nM Tris, pH 7.5, 1 M NaCl, 1 Mm EDTA) by heating the mixture at 100°C for 5 min and cooling to room temperature.

Genes	Forward	Reverse
GFP	GATCCCCGCAAGCTGACCCTGA AGTTCTTCAAGAGAGAACTTCA GGGTCAGCTTGCTTTTTGGAAA	AGCTTTTCCAAAAAGCAAGCTG ACCCTGAAGTTCTCTCTTGAAG AACTTCAGGGTCAGCTTGCGGG
TG2 (human)	GATCCCCGATGGGATCCTAGACA TCTTTCAAGAGAAGATGTCTAGG ATCCCATCTTTTTGGAAA	AGCTTTTCCAAAAAGATGGGAT CCTAGACATCTTCTCTTGAAAGA TGTCTAGGATCCCATCGGG

The annealed oligos were ligated into linearized pSuper plasmid (ref. 1) by HindIII and BglII enzyme digestion. The shRNA HaCaT cell lines were generated by co-transfection with pcDNA3 and pSuperGFP or pSuperTG2 vector. Cells were selected with G418 (1 mg/mL, Sigma-Aldrich, St Louis, MO, USA) for 1 week. All cell lines were regularly tested to exclude mycoplasma contamination.

QRT-PCR

Total RNA extraction and QRT-PCR were carried out using a CFX96TM Real-Time system (Bio-Rad, Hercules, CA, USA) as previously described (ref. 2).

Genes (human)	Forward	Reverse
TG1	TCATCTCTGCCATGGTGAACTCC	ACCAGACCAGTTCCCAATCAGG
TG2	AGAAGAGCGAAGGGACGTACTG	AGTCTACCACGTCGGCATTGAC
TG3	TGGCAGGTACATCAGCACCAAG	TGTCTTTCCTGGTCAGAGCCTTC
TG5	TCATCTTCGTGGTTGAAACTGGAC	ATTGGTCTCCAGCCAGGCAATC

Levels of mRNA were estimated by the $2^{-\Delta\Delta Ct}$ method and normalized to 36B4 levels.

Immunohistochemistry on cryosections

Mice were euthanized by CO_2 asphyxiation. The skin was harvested, embedded in Tissue-Tek OCT compound (Sakura Finetek, Inc., Torrance, CA, USA), and snap-frozen in liquid nitrogen. Frozen sections (10 μm) were cut from OCT-embedded tissues and fixed in cold acetone for 10 min and dried in air for 1 h. The sections were blocked with H_2O_2 and subsequently treated with 10% goat serum at room temperature for 20 min. They were then incubated with anti-loricrin polyclonal antibody (Abcam, Cambridge, MA, USA, ab85679), involucrin polyclonal antibody (Abcam, Cambridge, MA, USA, ab28057), and filaggrin polyclonal antibody (Abcam, Cambridge, MA, USA, ab24584) at 4°C for 18 h, followed by staining the proteins with Alexa Fluor 488-conjugated goat anti-rabbit IgG (H+L) secondary antibody (Molecular Probes, Inc., Eugene, OR, USA) in accordance with the manufacturer's instructions. The sections were mounted with fluorescent mounting medium (Dako North America, Inc., Carpinteria, CA, USA) and observed using a FluoView 1000 confocal microscope (Olympus, Tokyo, Japan).

Reference

1. Brummelkamp TR, Bernards R, Agami R. A system for stable expression of short interfering RNAs in mammalian cells. *Science* 2002; **296:** 550-3.
2. Lee SJ, Son YH, Lee KB, Lee JH, Kim HJ, Jeong EM *et al.* 4-n-butylresorcinol enhances proteolytic degradation of tyrosinase in B16F10 melanoma cells. *Int J Cosmet Sci* 2016; **39:** 248-255.

Supplementary Figures and Figure Legends

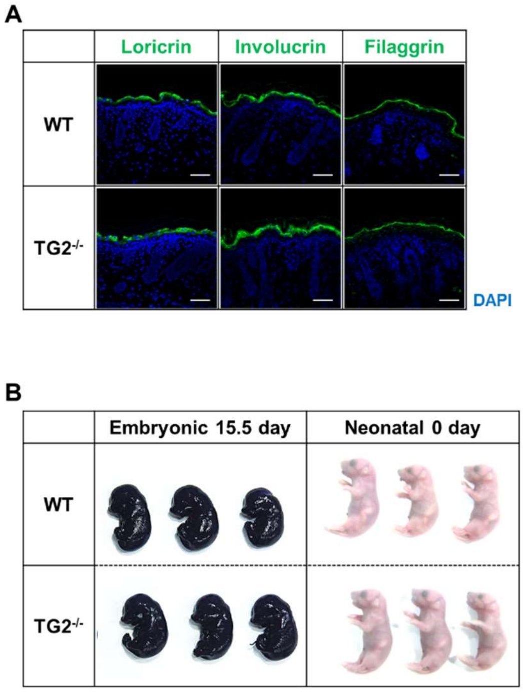

Supplementary Figure 1. TG2$^{-/-}$ mice show normal keratinocyte differentiation and skin barrier function. (**A**) Skin sections prepared from WT and TG2$^{-/-}$ neonates were immunostained with antibodies specific for loricrin, involucrin, and filaggrin. DAPI staining was used for localization of nuclei. Scale bar, 50 µm. (**B**) WT and TG2$^{-/-}$ neonates (birth day 0) or embryos (E15.5) were stained with toluidine blue.

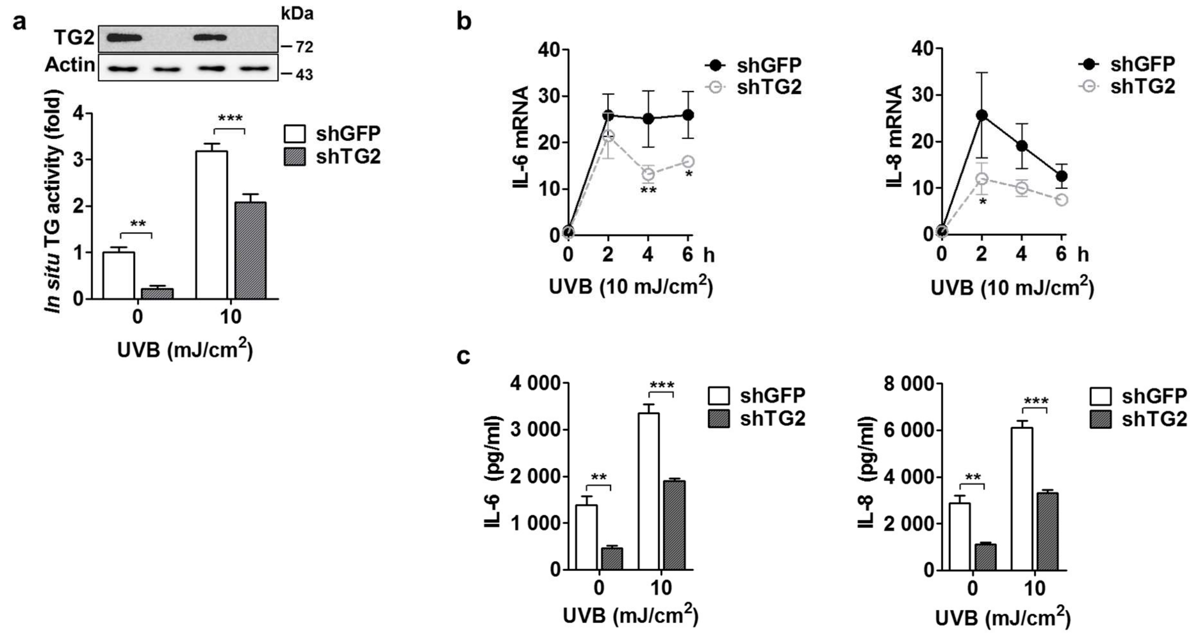

Supplementary Figure 2. Effect of TG2-knockdown on the expression of inflammatory cytokines in UV-irradiated HaCaT cells. (**A—C**) HaCaT cell line in which TG2 expression is downregulated was established by transfection of the shTG2 construct and selection. Cells were exposed to UV irradiation (10 mJ/cm^2). After 6 h, levels of *in situ* TG activity (**A**, n = 3), mRNA (**B**, n = 3), and secreted protein in culture media (**C**, n = 3) for IL-6 and IL-8 were measured by BP-incorporation assay, QRT-PCR assay, and cytometric bead array method, respectively. All data are represented as mean ± SEM. *, P<0.05; **, P<0.01; ***, P<0.001.

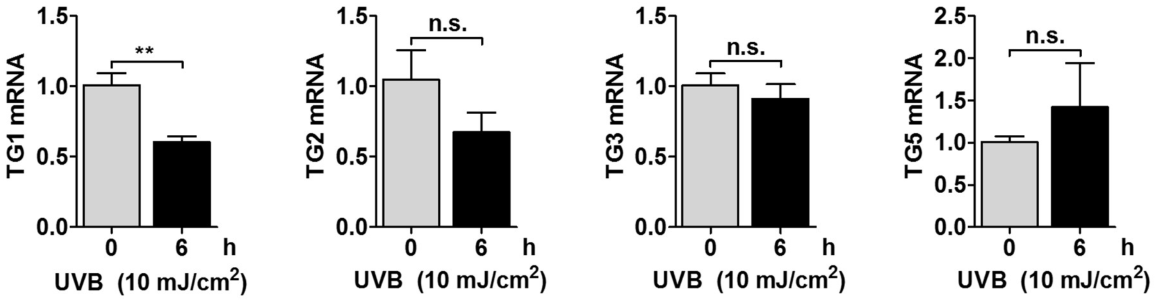

Supplementary Figure 3. Effect of UV irradiation on the expression of TG family enzymes in HaCaT cells. HaCaT cells were exposed to UV irradiation (10 mJ/cm^2), and mRNA levels of TG1, TG2, TG3, and TG5 were determined by QRT-PCR (n = 3). Data are represented as mean ± SEM. **, $P < 0.001$; n.s., not significant.
